# Combined effects of a topical fluoride treatment and 445 nm laser irradiation of enamel against a demineralization challenge: A light and electron microscopic *ex vivo* study

**DOI:** 10.1371/journal.pone.0237195

**Published:** 2020-08-07

**Authors:** Mohammed Abbood Al-Maliky, Matthias Frentzen, Jörg Meister

**Affiliations:** 1 Department of Periodontology, Operative and Preventive Dentistry, Dental Faculty, University of Bonn, Bonn, Germany; 2 Center of Applied Medical Laser Research and Biomedical Optics (AMLaReBO), Bonn University, Bonn, Germany; 3 Department of Biomedical Applications, Institute of Laser for Postgraduate Studies, University of Baghdad, Baghdad, Iraq; 4 Center of Dento-Maxillo-Facial Medicine, University of Bonn, Bonn, Germany; Danube Private University, AUSTRIA

## Abstract

This study investigated the caries-preventive effect of 445 nm laser radiation in combination with fluoride on the prevention of white spot lesions. Previously, several studies have indicated the ability of 488 nm argon ion laser irradiation to reduce early enamel demineralization. A diode laser (445 nm) could be an alternative technology for possible caries-preventive potential. Each sample of a group of seventeen caries-free bovine teeth was treated in four different ways on four different zones of the labial surface: control/no treatment (C), laser irradiation only (L) (0.3 W, 60 s and applied dose of 90 J/cm^2^), amine fluoride application only (10,000 ppm and pH 3.9) (F), and amine fluoride application followed by laser irradiation (FL). After treatment, the teeth were subjected to a demineralization solution (pH 4.3 for 48 h at 37 °C) to induce subsurface lesions. After sectioning, the teeth were examined by light microscopy. Three teeth were analyzed by scanning electron microscopy (SEM). The depths of the subsurface lesions in the C, L, F, and FL groups were 103.01 (± 13.04), 96.99 (± 14.51), 42.59 (± 17.13), and 24.35 (± 11.38) μm, respectively. The pairwise group comparison showed the following results: p < 0.001 for FL versus C, FL versus L, F versus C, and F versus L, p = 0.019 for FL versus F and p = 0.930 for L versus C. The SEM micrographs support the light-microscopic examination. The results of the current study have shown that using relatively low irradiation settings of 445 nm laser on fluoridated enamel may be effective for prevention of white spot lesions.

## Introduction

Globally, dental caries represents a primary public health problem. According to the Global Burden of Disease Study from 2005–2015, caries in permanent teeth was ranked the first prevalent chronic disease and was ranked twelfth for the deciduous dentition [1]. This condition affects eating and sleeping, and if left untreated, it can cause pain, abscesses and may be followed by systemic infection. One of the common causes for absence from work and school is related to dental decay [2].

Histologically, caries is characterized by loss of minerals from the enamel below the surface, where the surface is minimally affected. The removed minerals in the carious enamel leave subsurface porosities of approximately 25 vol% [3]. If the minerals loss reaches 50 vol%, a cavitation will occur [4].

To minimize the formation of dental decay, fluoride was introduced in toothpastes and mouthwashes and in the form of tablets for children. Fluoride interacts with enamel either by replacing and/or filling the hydroxyl group to form fluorohydroxyapatite with a lower solubility product or by formation of CaF_2_ deposits of the enamel surface and in the depth up to 40 μm from the surface [5, 6]. Due to the short time of topical fluoridation, it has been suggested that the formation of CaF_2_ is the major or possibly the only preventive mechanism of topical fluoridation [7]. Regardless, fluoridated apatite has been found below the CaF_2_ layer on the enamel surface to 20 μm of the subsurface [8]. After forming a surface coating layer, CaF_2_ can block the mineral diffusion channels around the enamel prisms, which reduces the permeability of enamel [9, 10].

Lasers have been tested for their caries-preventive potential since 1965 [11]. Laser irradiation action can be explained either by a photothermal modification of the inorganic and/or organic component of enamel or by a photochemical alteration of the enamel [12]. The photothermal effect of a CO_2_ laser (10600 nm and dose of 0.066 J/cm^2^) can lead to protein denaturation or to the decomposition of the carbonate component of the enamel, which can lead to decreases in enamel permeability and solubility, respectively [13]. Furthermore, the heating of enamel to its melting point (1200 °C) can lead to melting and fusion of enamel, providing a caries-resistant surface. The Er:YAG laser (2940 nm and energy density of 26.45 J/cm^2^) has been found to be effective in reducing enamel diffusion by 55% by its action on the organic component of enamel [14]. The photothermal interaction of a laser with enamel is usually accompanied by alteration of the enamel surface, which may be considered a limitation for the clinical use of this treatment modality [15]. On the other hand, argon ion (488 nm) and diode laser (810 nm) irradiation have been used in the photobiomodulation mode by applying low-power laser settings (0.25 W) and a long irradiation time up to 90 s alone or in combination with fluoride or photoabsorbing cream. In this mode of treatment, the enamel surface remains intact while achieving a caries-preventive effect [16, 17]. It has been suggested that argon ion laser can change the polarization of the enamel component, thus potentiating the retention and diffusion of fluoride to the inner layers of enamel and generates fluoride reservoir [18, 19]. In the majority of studies which employed the topical fluoride in combination with laser, the best results of fluoride uptake and reduction of enamel demineralization were observed when fluoride and laser were used together [20].

There are a few caries-preventive studies using lasers in the visible spectral range; lasers with wavelengths of 532, 633 and 670 nm showed a synergetic effect when combined with topical fluoride [21]. When a 445 nm laser was used in a recent polarized microscopy study, a significant preventive effect of a 9% decrease in lesion depth compared to that of the control was observed [22]. Previously, argon ion lasers (488 nm) have shown preventive effects both in vitro and in vivo and both alone and when combined with topical fluoridation [17, 18, 23–25]. However, when compared with diode lasers, the argon ion laser is less available for use in the dental market due to its high cost and large size. In the past, several studies have confirmed the ability of the 488 nm argon ion laser to reduce the demineralization of enamel [17, 23–25]. The mechanism of action of this laser was not clearly stated, and it was suggested that argon ion laser irradiation causes protein swelling leading to a reduced enamel permeability [26]. Based on these findings, the 445 nm laser, which was recently introduced to the dental market, could have a wavelength close enough to that of the argon ion laser, with a possible potential for caries prevention. This wavelength has a high absorption in pigmented tissues, low absorption in water and hydroxyapatite, increased scattering coefficient [27], high photon energy (eV), and photochemical control of protein interactions [28]. Accordingly, this study investigated the caries-preventive effect of the 445 nm laser in combination with fluoride.

This *ex vivo* study is aimed at investigating 445 nm laser irradiation and topical fluoride for a possible prevention of white spot enamel lesions using light and electron microscopy. The null hypothesis (H_0_) of the study is that 445 nm laser irradiation of fluoridated enamel is not effective for prevention of white spot lesions in bovine teeth.

## Materials and methods

### Sample preparation and grouping

**Fig 1:**
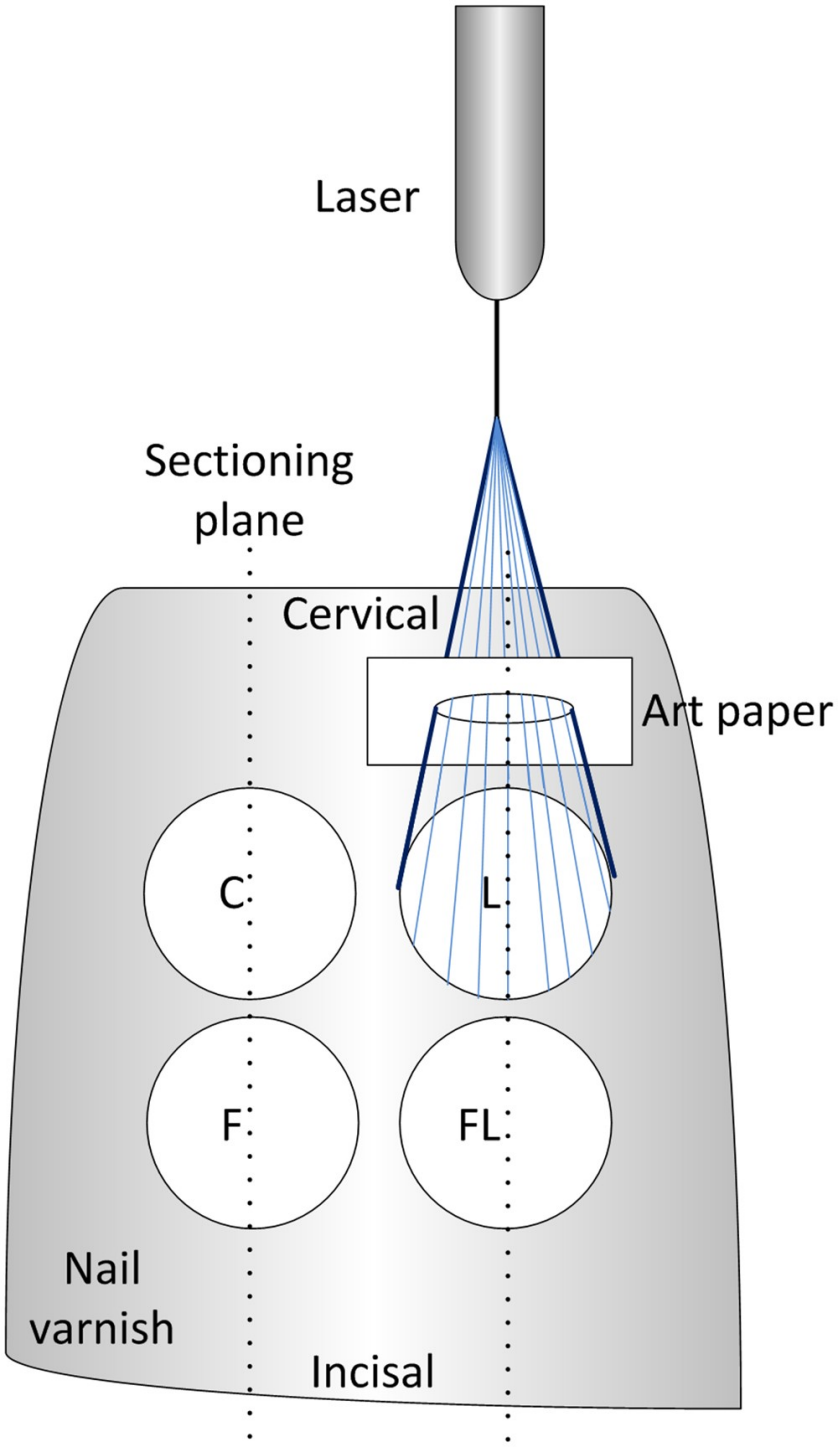
Schematic illustration of the treatment windows and sectioning planes. Control (C, no treatment), laser irradiation only (L), fluoride application only (F), and fluoride application followed by laser irradiation (FL) groups.

After approval by the institutional review board of the University of Bonn on the experimental protocol (01.17; bovine source: Rocholl, order#: 76685, Aglasterhausen, Germany), seventeen bovine teeth were stored in 0.9% NaCl with 0.001% sodium azide at 4 °C (Sodium azide: Fa. Merck, Darmstadt, Germany). The samples were polished with a nonfluoridated polishing paste (Cleanic RDA 27; Kerr, Biberach, Germany) and brushed with a brush bur for 10 s (Prophy Brush; Lüneburg Weimer, Frankfurt, Germany). Four windows (Ø = 5 mm) were marked on the labial surface of the teeth by covering the enamel surface with a nail varnish excluding these windows (Colour crush; The body shop, Dusseldorf, Germany). The treatment windows were assigned to control (C, no treatment), laser irradiation only (L), fluoride application only (F), and fluoride application followed by laser irradiation (FL) groups. To avoid possible influence from the window positions, each group was assigned to have an equal number of windows, which were located mesially, distally, gingivally, and incisally (Fig 1).

The lasing parameters of 0.3 W and 90 J/cm2 were based on a previous study and were verified by pretesting experiments of irradiation at 0.3 W for 10 and 60 s, which corresponds to applied doses of 10 and 90 J/cm^2^, respectively [22], These settings were adjusted to those previously used for argon ion laser (0.2–0.3 W and 10–100 J/cm^2^) [18, 25, 29].

### Treatment protocol

Sample treatment started with application of a topical fluoride solution (elmex*fluid*; CP GABA, Germany) containing 1% amine fluoride (10,000 ppm, pH 3.9, yellow colored solution) on the F and FL treatment windows. According to the manufacturer instructions, the fluoride fluid was kept in contact with the enamel surface for 3 min, followed by rinsing with 2.5 ml of distilled water. After being rinsed and left to dry, the L and FL treatment windows were irradiated with a prototype 445 nm laser (A.R.C. Laser, Nuremberg, Germany). The emitted laser radiation was guided through an optical fiber (Ø _outer diameter_ = 320 μm fiber) and was fixed with a universal laboratory clamp and kept at a distance of 15 mm perpendicular to the enamel surface. A verified no transmission art paper containing a window of 5 mm in diameter was placed on the enamel surface to avoid irradiation of the adjacent areas. The irradiation was conducted in continuous wave mode at 0.3 W for 60 s with an applied dose of 90 J/cm^2^ (Table 1) [22]. The power emitted from the laser device was calibrated using a power meter (LabMax Top) and a detector (PM-10), both from Coherent, CA, USA. At this time, the samples were placed in normal saline over the weekend for rehydration until being subjected to the demineralization solution for 48 h to induce subsurface demineralized lesions. The demineralization solution was composed of 2.0 mmol l–1 Ca, 2.0 mmol l–1 P, and 0.075 mol l–1 acetate buffer at pH 4.3 (Fa. Merck, Darmstadt, Germany) [30, 31]. Each sample was placed individually in 20 ml of the demineralization solution at 37 °C. The demineralization solution is undersaturated with respect to hydroxyapatite, so that the minerals are lost by diffusion/migration from enamel to the demineralization solution in order to reach equilibrium [32]. After demineralization, the samples were rinsed with distilled water to prepare them for evaluation of the treatment efficacy. Ten teeth were used for the histological examination with demineralization, four teeth treated as mentioned above without demineralization were used as a reference, and three teeth were used for SEM analysis after being subjected to the treatment and demineralization procedure.

**Table 1:** Parameters of laser irradiation.

Center Wavelength (nm)	445
Spectral bandwidth (nm)	+/- 3
Operating mode	Continuous wave
Average radiant power (W)	0.3
Polarization	Random
Aperture diameter (cm)	0.32
Power density at aperture (W cm-^2^)	3.75
Beam area at target (cm^2^)	0.2
Irradiance (W cm^-2^)	1.5
Exposure time (s)	60
Radiant exposure (J cm^_2^)	90
Energy density at aperture (J cm-^2^)	225
Radiant energy (J)	18
Application technique	Stationary

### Histological analysis

For the histological examination, the teeth need to be prepared for sectioning, and this requires fixation, dehydration, and infiltration of the teeth by resin. This process is done in a way such that the water inside the samples is removed gradually and replaced by resin. Dehydration is conducted by immersion of the teeth in series of increasing alcohol concentrations.

**Fig 2:**
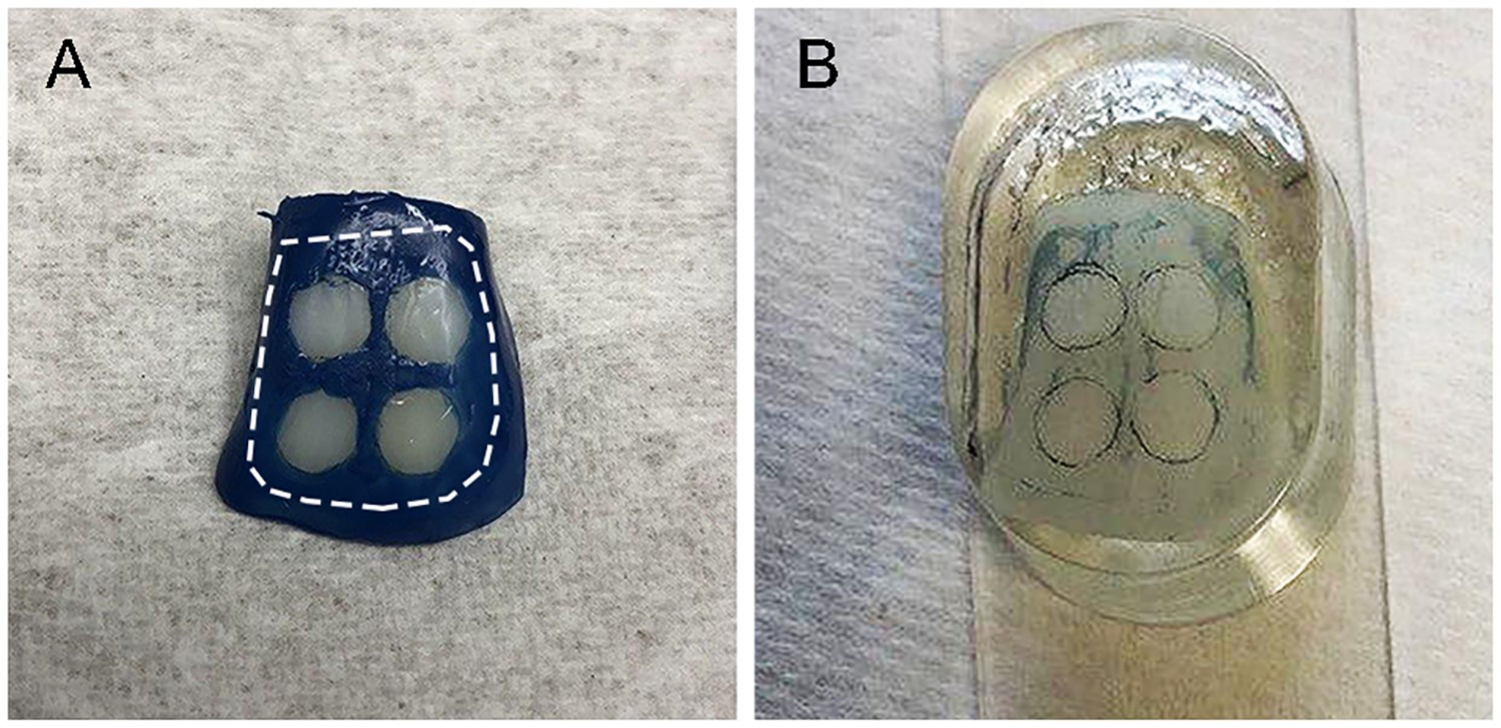
Removal of nail varnish before samples processing for histological preparation. A = treated and lased sample with nail varnish is covering all the tooth except the four treatment windows: the nail varnish was planned to be removed from all around the tooth until the white dotted line by acetone, then polished with pumice. B = tooth embedded in PMMA with the nail varnish was removed: the remaining part of the nail varnish around the treatment windows encircled by the white dotted line was detached from the tooth during the alcohol dehydration process.

**Fig 3:**
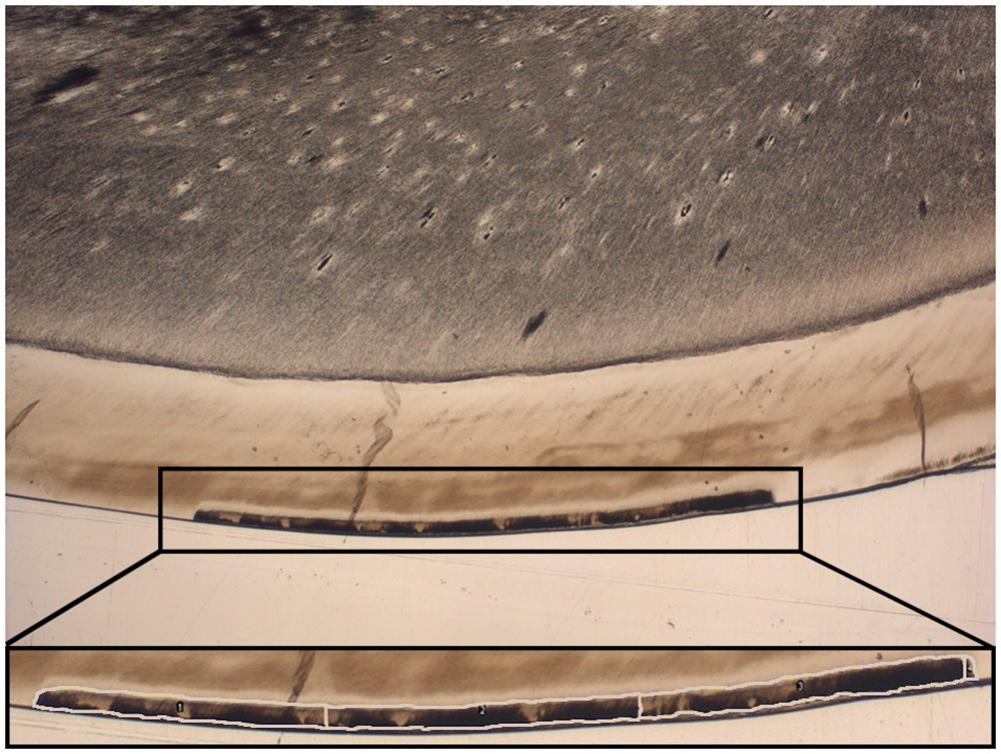
Measurement of the lesion body area (mm^2^) using ImageJ software. The demineralized area is marked by the white line.

**Fig 4:**
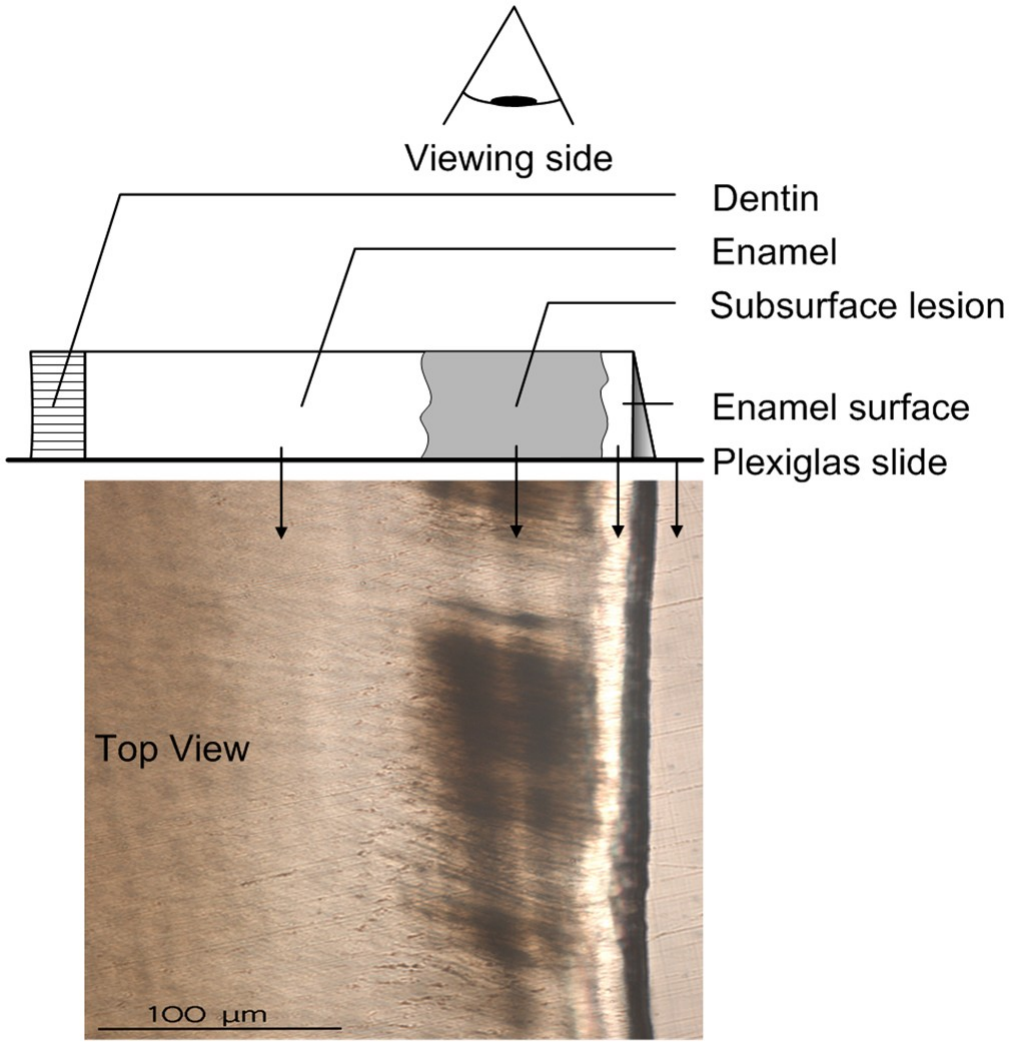
Illustration of the histological landmarks of the demineralized samples.

To avoid interference with the sample fixation and infiltration process, the nail varnish was removed with acetone (Ebelen; dm-drogerie markt, Karlsruhe, Germany) using a tweezer and cotton pellet, and the teeth were polished with fluoride free powder of pumice mixed with normal saline using brush bur mounted in a low speed handpiece, except the treatment windows (Fig 2). Afterward, the treated and both demineralized and nondemineralized teeth were fixed in 4% formaldehyde (Fa. Merck, Darmstadt, Germany), rinsed with distilled water, and dehydrated with increasing concentrations of 70–100% ethanol alcohol (Ethylalcohol; Werner Hofmann, Dusseldorf. Germany) under agitation. After dehydration, the teeth were infiltrated by a two-step immersion in resin for 10 days each under vacuum. The first step of the infiltration was composed of a 1:1 combination of polymethylmethacrylate (PMMA) (Technovit 7200 VLC; Heraeus Kulzer, Hanau, Germany) and 2-hydroxyethyl methacrylate (GMA) (Sigma-Aldrich, St. Louis, USA), and in the second step, the teeth were embedded in 100% PMMA. After infiltration, the samples were embedded in PMMA and cured under blue and yellow lights for 4 h each, and the obtained blocks were sectioned with a precision sectioning machine (300 CPV; EXAKT, Norderstedt, Germany) to a thickness of 300–400 μm. These sections were glued with resin (Technovit 7210 VLC; Heraeus Kulzer, Hanau, Germany) to Plexiglas slides and polished until obtaining 90 (± 10) μm-thick slices using a microgrinding machine and #4000 carbide paper (400 CS; EXAKT, Norderstedt, Germany). Sections were evaluated using a light microscope at a magnification of 16×–400× (Dialux 20 EB; Leica Microsystems, Wetzlar, Germany). The micrographs were obtained using the microscope camera (DFC420 C; Leica Microsystems), and the photos were quantified by measuring the area of the body of the lesions in square millimeters and dividing it by the respective lesion length to obtain the average lesion depth using analytical software (ImageJ 1.51K; NIH, USA) [33] (Figs 3 and 4).

### SEM analysis

After treatment and demineralization as mentioned in the treatment protocol above, the teeth were washed with distilled water, glued to Plexiglas slides from the mesial or distal side with a cold cure resin (Technovit 4000; Heraeus Kulzer, Hanau, Germany) and sectioned (~ 400 μm-thick slices) incisocervically using a precision sectioning machine (300 CPV; EXAKT, Norderstedt, Germany) in the center of the treatment windows. The obtained slices were polished with #500 and #1200 carbide paper (EXAKT, Norderstedt, Germany) rinsed with distilled water and acid-etched with 35% phosphoric acid gel (Vococid; Voco, Cuxhaven, Germany) for 10 s to remove the smear layer, and the acid etch was removed by rinsing with distilled water. After fixation by 4% paraformaldehyde (PFA) in phosphate-buffered saline (PBS) (Fa. Merck, Darmstadt, Germany), the teeth were immersed in serial ethanol alcohol concentrations for dehydration (Ethylalcohol; Werner Hofmann, Dusseldorf. Germany); the dehydration proceeded for 1 h in each concentration of 2× 30, 2× 50, and 2× 70% ethanol. Then, the teeth were placed in 80% ethanol overnight followed by 24 h of 90, 95, and 100% concentrations each and then were left overnight to dry. Next, the teeth were sputtered with platinum (Platinum Target Scancoat 6; Edwards, Lomma, Sweden) for examination by SEM (XL 30; Philips, Eindhoeven, Netherlands) operated at 25 kV. The selected area for examination was the cross-section of the enamel including the surface-cross-section line angle.

### Statistical analysis

For the statistical and descriptive statistics, IBM SPSS (Ver. 21.0; IBM, Armonk, NY, USA) was used. The Shapiro-Wilk test was used to check the data normal distribution. To test the treatment efficacy, the data were analyzed using the repeated measures analysis of variance (RM-ANOVA) with Sidak confidence interval adjustment followed by pairwise group means comparisons. The significance limit was set to *p* = 0.05.

## Results

### Histological quantitative and qualitative analysis

**Fig 5:**
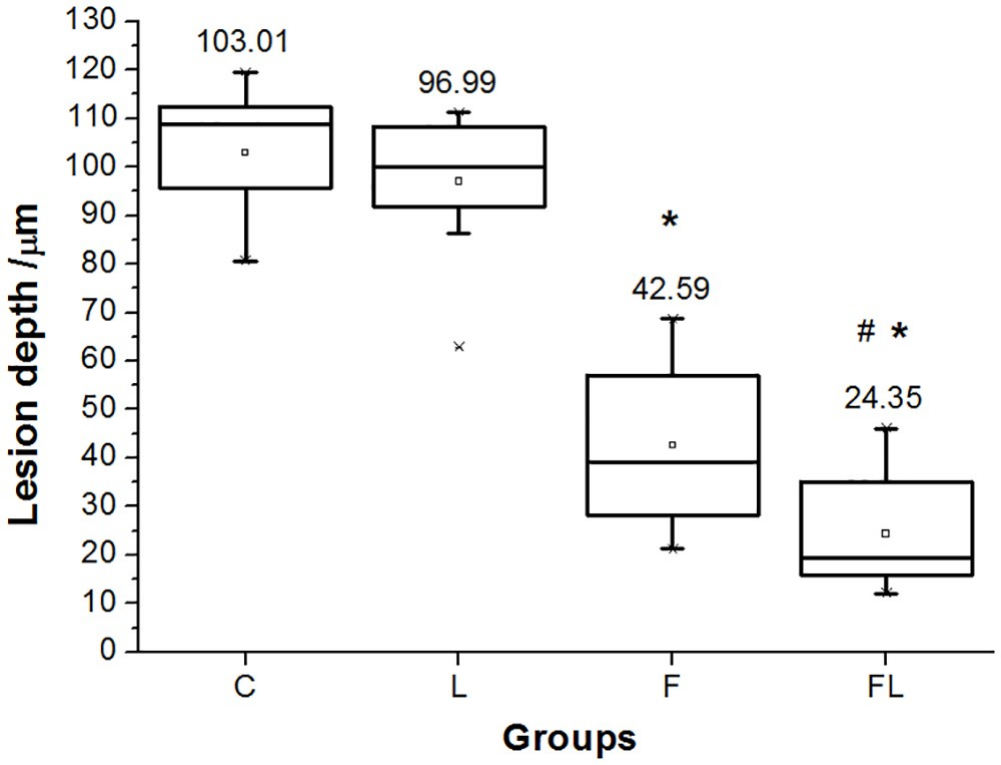
Box and whisker plot of the depth of the lesion bodies. No treatment/control (C), laser (L), fluoride (F) and fluoride followed by laser (FL) treatment groups. The (▫) symbol inside the box = mean, the horizontal line inside the box = median, and the end of the upper and lower whiskers of the box = maximum and the minimum that are not outliers, respectively. The (˟) symbol outside the whisker = outlier. The numbers above the boxes = groups’ mean values. * = significant difference compared to all other groups, and the (#) mark represents a significant difference when compared with the F group at *p* < 0.05, n = 10.

**Fig 6:**
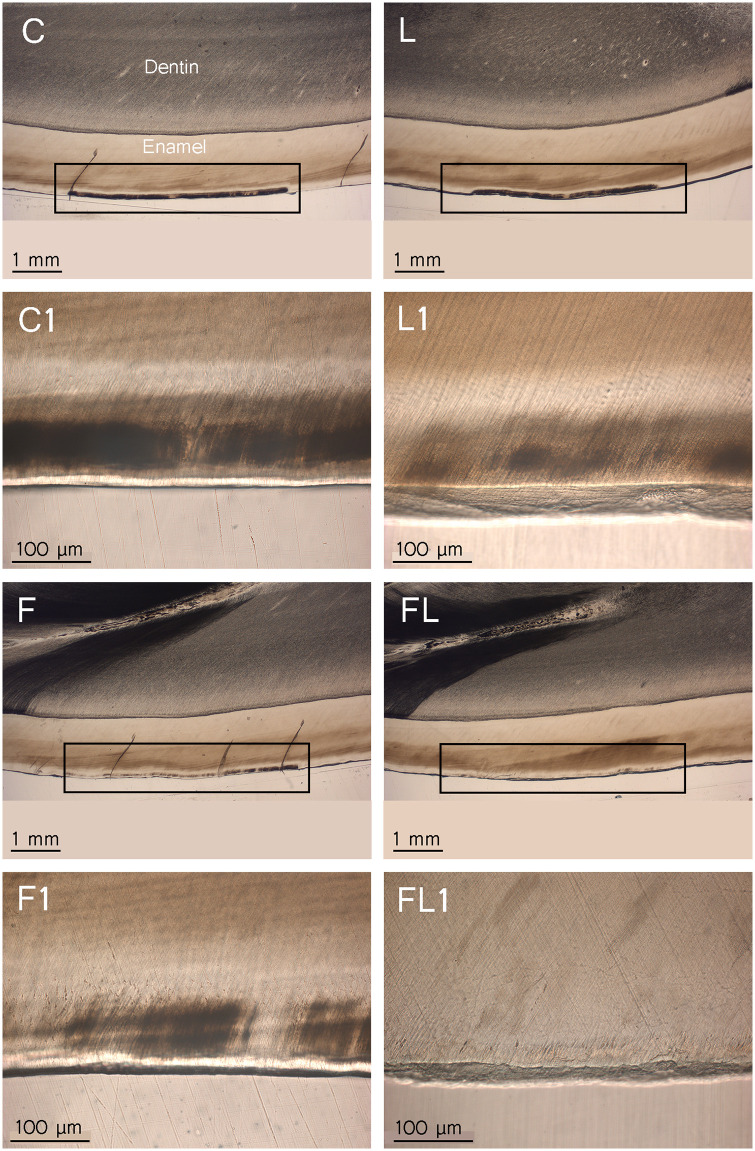
Cross-sectional histological photographs of the paired representative lesions, (n = 10). C = control, L = laser, F = fluoride and FL = fluoride followed by laser. The frame marks the area of treatment. The second and fourth rows are higher magnifications (250x) of the first and third rows (16x), respectively.

**Fig 7:**
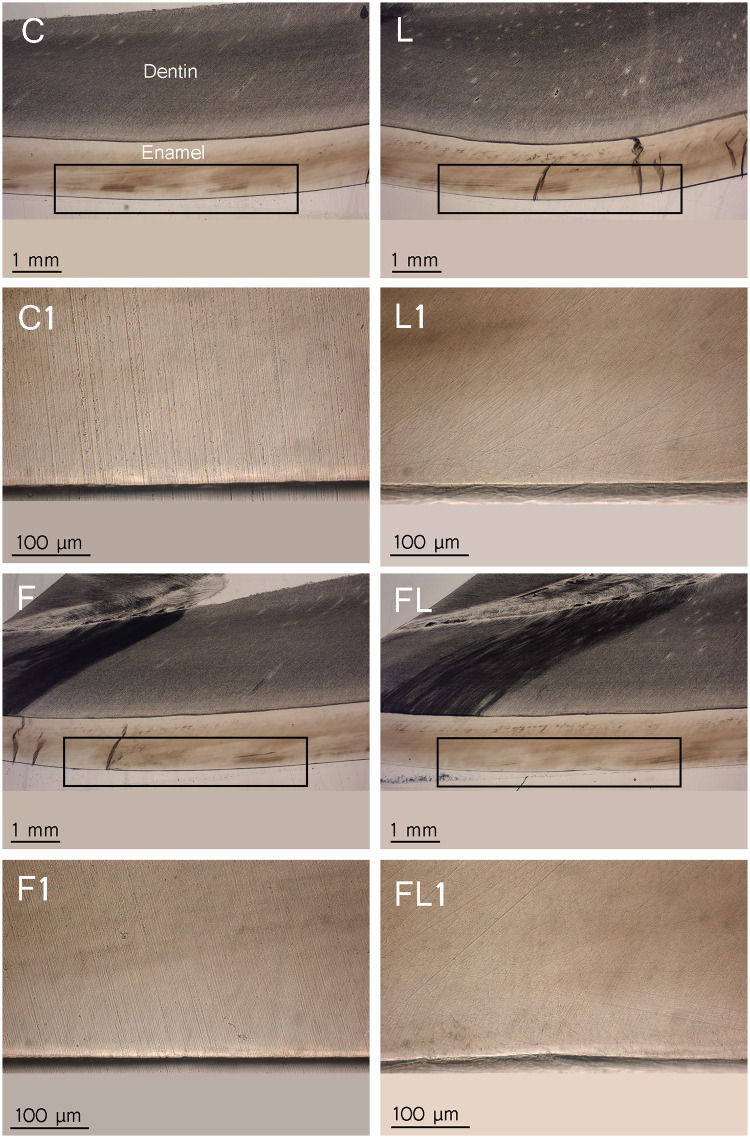
Cross-sectional histological photographs of the paired representative nondemineralized samples (n = 4). C = control, L = laser, F = fluoride and FL = fluoride followed by laser. The frame marks the area of treatment. The second and fourth rows are magnifications (250x) of the first and third rows (16x), respectively.

The descriptive statics showed a normal distribution of the data by the mean of the Shapiro-Wilk test. The mean and standard deviation of the depth of the lesion bodies for the C, L, F, and FL groups were 103.01 (± 13.04), 96.99 (± 14.51), 42.59 (± 17.13), and 24.35 (± 11.38) μm, respectively (Fig 5). RM-ANOVA testing showed a significant treatment effect with *F*_(3, 27)_ = 115.63, *p* < 0.001. The effect of the pairwise comparisons of the mean showed that all cross-comparisons between the treatment groups were significant except the L group compared to the C group. The *p* values of these comparisons were *p* < 0.001 for FL versus C, FL versus L, F versus C, and F versus L, and it was *p* = 0.019 for FL versus F and *p* = 0.930 for L versus C (Table 2). The decrease in the depth of the lesion bodies for the L, F, and FL groups compared with the C group was 6, 58, and 77%, respectively. Regarding the quality assessment of the subsurface lesions, the lesions of the L and FL groups revealed a lighter black color than that of the C and F groups, respectively during the histological examination, thus indicating a lower degree of demineralization in enamel (Fig 6). Histological examination of the treatment groups without demineralization was also performed to be used as reference for the demineralized groups. The nondemineralized samples showed normal histological findings with no subsurface lesions (Fig 7).

**Table 2:** Pairwise comparison statistics (*p* value) of the mean depths (μm) of the lesion bodies.

Groups	C 103.01 (± 13.04)	L 96.99 (± 14.51)	F 42.59 (± 17.13)	FL 24.35 (± 11.38)
C 103.01 (± 13.04)	-	0.930	<0.001*	<0.001*
L 96.99 (± 14.51)	0.930	-	<0.001*	<0.001*
F 42.59 (± 17.13)	<0.001*	<0.001*	-	0.019*
FL 24.35 (± 11.38)	<0.001*	<0.001*	0.019*	-

* The mean difference is significant at the 0.05 level.

### SEM analysis

**Fig 8:**
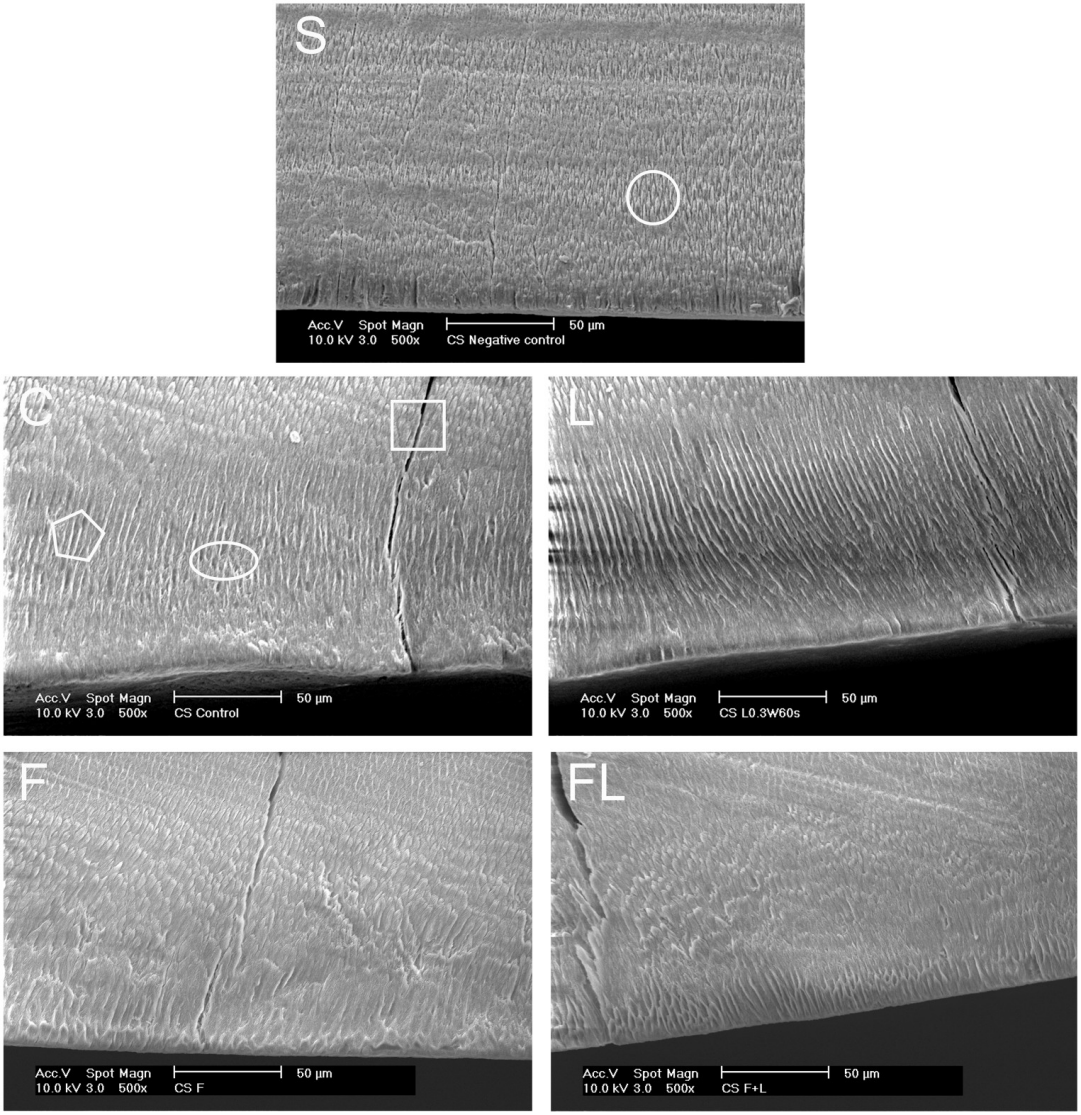
Cross-sectional SEM micrographs (500x) (n = 3). S = sound enamel, C = control, L = laser, F = fluoride and FL = fluoride followed by laser. The circle shape = normal enamel rod appearance; the polygon shape = dissolution of the enamel crystals; the ellipse shape = enamel subsurface porosity; and the square shape = crack resulting from the machine vacuum.

The cross-section of the sound enamel was also included in the evaluation process as a negative control (no treatment). Fig 8S shows a normal appearance of the enamel rods and the inter-rod enamel until the enamel surface-cross section line angle. In the untreated demineralized enamel, the subsurface enamel crystals were dissolved by the action of the demineralization solution, and the area of dissolution showed signs of severe porosity, with the enamel rods either completely dissolved or decreased in size, leaving spaces between the inter-rod enamel (Fig 8C). In the lased and demineralized group, the topography of the cross-section ranged from a similar appearance to that of the control group to an appearance with lower dissolution and porosity zones (Fig 8L). In the SEM results of the F group, the dissolution zone disappeared, with minimal signs of porosity, and the area of the lesion body could be demarcated (Fig 8F). Finally, the SEM results of the FL group showed minimal signs of demineralization in the enamel, and the subsurface lesion was not well defined, lacking dissolution or porosity (Fig 8FL).

## Discussion

The H_0_ of the study was rejected as the 445 nm laser irradiation of fluoridated enamel was effective in prevention of white spot lesions in bovine enamel. When enamel fluoridation was followed by 445 nm laser irradiation, the histological examination showed a resistance to acid demineralization of 77% compared with that of the control group, and this result was significant when compared to the enamel fluoridation only group. Laser irradiation alone reduced enamel demineralization by 6% compared with that of the control group. The SEM results are consistent with the histological analysis. In addition to the histological measurement of the subsurface demineralization, the qualitative assessment showed that the demineralized area of the F group is darker than that of the FL group (Fig 6).

The factors that govern the interaction of fluoride with the tooth surface are the time of application, fluoride concertation, and pH of the topical fluoride treatment [34]. Additionally, the absorbed 445 nm laser light by the topical amine fluoride is 22% (0.241 a.u.), and the elevation of surface temperature of enamel to ΔT of 290.16 °K during lasing may accelerate the rate of the chemical reaction between the fluoride and enamel [22]. By increasing the CaF_2_ formation both on the enamel surface and up to 40 μm of the subsurface, the diffusion pathways of the enamel will become occluded, leading to reduced dissolution of the enamel [6]. The histological observation of this study supports the assumption of the reduced permeability, and this is because the F group and to a greater extent the FL group showed reduced lesion bodies. Otherwise, if the permeability was not reduced, we would expect a severe demineralization beyond the first 40 μm of the fluoridated enamel, and this was not seen in the current study. The preventive effect of the L group in this study using the 445 nm laser (6%) is lower than those achieved using the 488 nm argon ion laser (15–41%), and this could be explained by the difference in the wavelengths between these lasers [35, 36]. This difference may lead to different absorption properties of the laser light by the tissue chromophores, which in turn leads to variations in the preventive effectiveness. Although it was concluded that there is no significant difference if the fluoride is applied before or after laser irradiation, after conducting pre-experiments and based on previous studies, the fluoride was applied prior to laser irradiation [22, 21]. When treatment efficacy of the fluoride plus laser of this histology study (77%) are compared with that of a recent 445 nm polarized light microscopy (PLM) study (28%) for prevention of white spot lesions, it shows a higher effectiveness [22]; although both studies irradiated bovine teeth with similar laser settings, the variation in the effectiveness of treatment could be explained by the difference in the investigation methodology. In the mentioned PLM study, samples’ cracking has restricted the thickness of the samples to a minimum of 250 μm, and this thickness has limited the magnification to a maximum power of 16×: although this test gave an overview for the treatment efficacy through measurement of lesions depth, little details were obtained about the severity of lesions between the different treatment groups. This may be explained by the presence of less affected lesions for the FL and F groups that have been seen as a demineralization under the polarized microscope due to the increased light scattering resulted from the use of thicker samples. During the current study, histological preparation offered a thin samples thickness of 90 μm with a possibility of high magnification up to 400×, then the FL (77%) and to a less extent F (58%) groups have shown less affected enamel surface and short subsurface lesions compared with the C group due to the decreased light scattering when it passes through thinner samples. This may has been led to the elimination of possible false positive recordings for the lesion depths reported during the PLM investigation as a consequence of the use of thicker samples. This observation was not reported for the control or the laser only groups due to the presence of high degree of demineralization, thus samples thickness will have less influence.

The SEM morphological observation further supports the histological examination. The demineralization in the C and L groups was more obvious than that in the F and the FL groups. If the lesion body is slight like those of the F and FL groups, the SEM examination will have a lower ability to clearly show the demineralization than the histological examination will. On the other hand, the histological microscopy technique gives information about the whole thickness of the specimen under analysis; furthermore, it represents a direct in vitro method for caries observation and quantification compared with the other indirect methods, which measure, for example, the decrease in the microhardness or fluorescence of enamel. As the observed demineralization in this study has irregular margins and is not extended to the enamel surface, the area of the lesion bodies was divided by the respective length of each lesion to give the average lesion depth [33] (Fig 4).

Due to the lack of data for the 445 nm laser parameters for caries prevention, the used irradiation settings were based on a recent polarized microscopy study [22] on different teeth, and they fall in the range used for the argon ion laser [17, 29]. These parameters caused neither histological changes in enamel nor signs of thermal damage when investigated before the demineralization challenge (Fig 7). Furthermore, these settings were safe for the dental pulp of human molar teeth with an average increase in the pulpal temperature of 275.28 °K [22].

The used demineralization only protocol in the current study was chose to make the comparison with previous argon ion studies more valid, that the demineralization protocols used in these studies were the demineralization only without the remineralization [35, 36]. Additionally, although pH-cycling models are close to the clinical situation it still different that the caries formed inside the oral cavity is caused by multiple intermittent episodes of upwards and downwards of pH during 24 h, and this was indicated to accelerate the transformation of reaction products formed [32]. Both demineralization only and pH-cycling protocols were used in the literature to investigate treatment efficacy, and in 2012 Tavaris et al. have found that there was no statistical difference between them in the lesions area recorded in the control samples (0.25 versus 0.18 mm^2^) and argon ion irradiated samples (0.14 versus 0.07 mm^2^) [17].

For standardization, bovine teeth were used to avoid previous fluoridation. Additionally, the treatment was conducted on natural enamel surface without flattening to simulate the in vivo conditions [37]. Bovine enamel is readily available, and has found to have a close behavior to that of human enamel regarding biofilm cariogenicity and lesion severity [38]. However, bovine enamel has shown to have a higher hardness, larger crystal size, and faster rate of lesion progression (1.4:1) than those of human enamel [39–41]. Accordingly, the results of the present study which employed standard caries testing methods on bovine teeth may be considered as initial indicator for the treatment efficacy: they need to be verified by future studies on human teeth implementing microhardness or micro-CT to be more relevant clinically. In the current study, the laser irradiation was done on the labial surface of the tooth and could be applied to the occlusal surface. As dental caries remains one of the most prevalent chronic diseases [1], the clinical rational for this modality is the introduction of a patient-independent technique for prevention of early caries formation. Which is best suited for children and adolescents. However, a general limitation for laser treatment clinically is that irradiation of the inaccessible areas such as proximal tooth surfaces is still a challenge. In the oral environment, the fluoride could exhibit antibacterial action [8]. The use of lasers in sub-ablative irradiation settings found to be effective in preventing enamel demineralization when associated with topical fluoride products with conclusions analogous to those found in this study [42].

## Conclusion

Under the limitations of this *ex vivo* study, the results of the current study have shown that using relatively low irradiation settings of 445 nm laser on fluoridated enamel may be effective for prevention of white spot demineralization. Further research is recommended in this field to reach appropriate noninvasive patient independent caries preventive methods.

## Supporting information

S1 File Raw data set for the histological test. (DOCX)
