# Supplementary material for: Combined effects of a topical fluoride treatment and 445 nm laser irradiation of enamel against a demineralization challenge: A light and electron microscopic ex vivo study
Source: PLoS One. 2020 Aug 7;15(8):e0237195. doi: 10.1371/journal.pone.0237195 (PMC7413416; doi:10.1371/journal.pone.0237195)
Supplement: S1 File — (DOCX) [file pone.0237195.s001.docx]

S1 File. Raw data set of the depths (µm) of the lesion bodies.

| n | Control | Laser | Fluoride | Fluoride + Laser |
| --- | --- | --- | --- | --- |
| 1 | 97.27 | 108.94 | 28.24 | 21.76 |
| 2 | 112.51 | 102.06 | 62.69 | 15.95 |
| 3 | 114.21 | 98.25 | 68.66 | 46.15 |
| 4 | 110.17 | 62.82 | 24.53 | 12.11 |
| 5 | 95.77 | 86.35 | 43.92 | 16.46 |
| 6 | 80.67 | 105.65 | 34.47 | 15.21 |
| 7 | 107.88 | 108.34 | 54.59 | 28.11 |
| 8 | 109.66 | 94.44 | 21.24 | 16.80 |
| 9 | 119.56 | 111.33 | 57.11 | 35.95 |
| 10 | 82.42 | 91.77 | 30.46 | 34.96 |
